# Draft Genome Sequences of Five Shiga Toxin-Producing *Escherichia coli* Isolates Harboring the New and Recently Described Subtilase Cytotoxin Allelic Variant *subAB_2-3_*

**DOI:** 10.1128/genomeA.01582-16

**Published:** 2017-02-23

**Authors:** Taurai Tasara, Lisa Fierz, Jochen Klumpp, Herbert Schmidt, Roger Stephan

**Affiliations:** a Institute for Food Safety and Hygiene, Vetsuisse Faculty University of Zurich, Zürich, Switzerland; b Institute of Food, Nutrition and Health, ETH Zürich, Zürich, Switzerland; c Department of Food Microbiology and Hygiene, Institute of Food Science and Biotechnology, University of Hohenheim, Stuttgart, Germany

## Abstract

We present here the draft genome sequences of five Shiga toxin-producing *Escherichia coli* (STEC) strains which tested positive in a primary *subAB* screening. Assembly and annotation of the draft genomes revealed that all strains harbored the recently described allelic variant *subAB*_*2-3*_. Based on the sequence data, primers were designed to identify and differentiate this variant.

## GENOME ANNOUNCEMENT

The subtilase cytotoxin (SubAB) is an AB_5_ cytotoxin identified in certain Shiga toxin-producing *Escherichia coli* (STEC) strains, in particular in locus of enterocyte effacement (LEE)-negative strains, and was originally discovered to be encoded by *subAB*_*1*_ on the large conjugative virulence plasmid pO113 (1). In addition to *subAB*_*1*_, two chromosomal variants, *subAB*_*2-1*_, located on the pathogenicity island (PAI) SE-PAI (2, 3), and its allelic variant *subAB*_*2-2*_, contained on an outer membrane efflux protein (OEP) locus, have been described (4). Recently, we described an isolate (*E. coli* strain 48) originating from a roe deer and testing positive in the primary *subAB* screening but which was negative in subsequent subtyping for *subAB* variants (5). This strain harbored a new *subAB*_*2*_ allelic variant that has been designated *subAB*_*2-3*_ and was associated with a gene predicted to encode a hypothetical protein of yet-unknown function, which is located 527 bp upstream of the *subAB* locus. The new genetic location of this *subAB* operon did not show any sequence similarity to those associated with *subAB*_*2-1*_ and *subAB*_*2-2*_ alleles. As such, this allele cannot be typed using the current *subAB* typing primer sets that include binding targets located in terpenoid indole alkaloid (Tia)- and OEP-encoding genes.

Here, we describe the draft genome sequences of five new STEC strains (strain* E. coli* N11-1317, a clinical human isolate, and strains *E. coli* 113, *E. coli* 117, *E. coli* 256, *E. coli* 453 isolated from healthy reindeers at slaughter) which tested positive in the primary *subAB* screening but which were negative in subsequent subtyping for *subAB* variants. Genomic DNA was isolated from these strains and sequenced using Pacific Biosciences single-molecule real-time sequencing (SMRT) technology at the Functional Genomics Centre of the University of Zurich. The obtained sequences were assembled *de novo* using the SMRT Analysis 2.3.0 software and annotated using the NCBI Prokaryotic Genome Automatic Annotation Pipeline (http://www.ncbi.nlm.nih.gov/genome/annotation_prok/). Draft genomes that were determined in the five strains, including the chromosomes and plasmids, ranged from 5.38 Mb to 6.18 Mb, with each genome containing 5,278 to 6,046 coding sequences (Table 1).

**TABLE 1:** Sequenced *subAB_2-3_*-positive strains and their genome accession numbers

*E. coli* strain	Source	Genome size (Mb)	No. of coding sequences	Accession no.
N11-1317	Human	5.63	5,465	MPGQ00000000
453	Reindeer	5.79	5,771	MPGR00000000
113	Reindeer	6.18	6,046	MPGP00000000
117	Reindeer	5.38	5,278	MPGS00000000
256	Reindeer	5.54	5,425	MPGT00000000

An analysis of the draft genome sequences revealed that in all five strains, the *subAB*_2-3_ operon was chromosomally located. Sequence comparison with the *E. coli* 48 genome (accession no. JPQG00000000) showed that all five strains harbored the *subAB*_*2-3*_ allelic variant. Within all five strains, the *subAB*_*2-3*_ operon is located between two genes that encode hypothetical proteins of yet-unknown functions and correspond to JD73_15220 and JD73_15240 in the *E. coli* 48 genome. Based on these sequence data, we designed a new primer, subAB2-3rv (GAGGCGACTAATGAAGAATTAA), which binds within the gene for JD73_15240. Use of this primer in combination with a previously described subAB_out primer (GAATCAACAACAGATACGAC [4]) allows the identification of the *subAB*_2-3_ variant based on a 943-bp PCR amplification product. This new primer, subAB2-3rv, can thus also be included in the current *subAB* PCR subtyping scheme to identify the *subAB*_2-3_ variant.

### Accession number(s).

These whole-genome shotgun projects for the five strains have been deposited in GenBank under accession numbers provided in Table 1. The versions described in this paper are the first versions.
